# An Exact Expression to Calculate the Derivatives of Position-Dependent Observables in Molecular Simulations with Flexible Constraints

**DOI:** 10.1371/journal.pone.0024563

**Published:** 2011-09-12

**Authors:** Pablo Echenique, Claudio N. Cavasotto, Monica De Marco, Pablo Garca-Risueño, J.L. Alonso

**Affiliations:** 1 Instituto de Química Física “Rocasolano”, Consejo Superior de Investigaciones Científicas (CSIC), Madrid, Spain; 2 Instituto de Biocomputación y Física de Sistemas Complejos (BIFI), Universidad de Zaragoza, Zaragoza, Spain; 3 Departamento de Física Teórica, Universidad de Zaragoza, Zaragoza, Spain; 4 Unidad Asociada IQFR-BIFI, Zaragoza, Spai; 5 School of Biomedical Informatics, University of Texas Health Science Center at Houston, Houston, Texas, United States of America; Aston University, United Kingdom

## Abstract

In this work, we introduce an algorithm to compute the derivatives of physical observables along the constrained subspace when flexible constraints are imposed on the system (i.e., constraints in which the constrained coordinates are fixed to configuration-dependent values). The presented scheme is exact, it does not contain any tunable parameter, and it only requires the calculation and inversion of a sub-block of the Hessian matrix of second derivatives of the function through which the constraints are defined. We also present a practical application to the case in which the sought observables are the Euclidean coordinates of complex molecular systems, and the function whose minimization defines the flexible constraints is the potential energy. Finally, and in order to validate the method, which, as far as we are aware, is the first of its kind in the literature, we compare it to the natural and straightforward finite-differences approach in a toy system and in three molecules of biological relevance: methanol, N-methyl-acetamide and a tri-glycine peptide.

## Introduction

In the theoretical and computational modeling of physical systems, including but not limited to condensed-matter materials [1], fluids [2], and biological molecules [3], it is very common to appeal to the concept of *constraints*. When a given quantity related to the system under study is constrained, it is not allowed to depend explicitly on time (or on any other parameter that describes the evolution of the system in the problem at hand). Instead, a constrained quantity is either set to a constant value (*hard* or *rigid* constraints) or to a function of the rest of degrees of freedom (*flexible*, *elastic* or *soft* constraints); in such a way that, if it depends on time, it does so through the latter and not in an explicit manner.

The imposition of constraints is useful in a wide variety of contexts in the fields of computational physics and chemistry: For example, we can use constraints to maintain an exact symmetry of the equations of motion; like in Car-Parrinello molecular dynamics (MD) [4], where the time-dependent Kohn-Sham orbitals need to be orthonormal along the time evolution of the quantum-classical system, a requirement that can be fulfilled by imposing constraints over their scalar product [5]. In a different context, we can use constraints, as in the Blue Moon Ensemble technique [6], to fix some macroscopic, representative degrees of freedom of molecular systems (normally called *reaction coordinates*), in order to be able to compute free energy profiles along them that would take an unfeasibly long time if we used an unconstrained simulation. Probably the most common application of the idea of constraints, and the one that will be mainly discussed in this work, appears when we fix the fastest, hardest degrees of freedom of molecular systems, such as bond lengths or bond angles, in order to allow for larger time-steps in MD simulations [7], [8].

In any of these cases (assuming that the dimensions of the spaces involved are all finite) the imposition of constraints can be described in the following way: If the state of the system is parameterized by a given set of coordinates 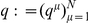, spanning the *whole space*, 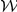, and the associated momenta 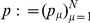, a given *constrained subspace*, 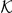, of dimension 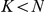, can be defined by giving a set of 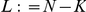 independent relations among the coordinates (In this work, we will only deal with holonomic, scleronomous constraints, i.e., those that are independent both of the momenta and (explicitly) of time.):
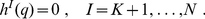
(1)


The condition of these constraints being independent amounts to asking the set of 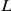 vectors of 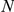 components
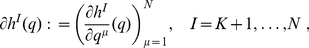
(2)to be linearly independent at the relevant points 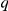 satisfying (1), and it means that 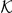 is a manifold of constant dimension in these points, which are called *regular*. Moreover, this independence condition allows, in the vicinity of each point 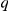 and by virtue of the Implicit Function Theorem [9], [10], to (formally) solve (1) for 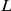 of the coordinates, which we arbitrarily place at the end of 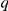, splitting the original set as 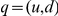, with 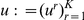 and 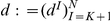. Then, in the vicinity of each point 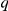 satisfying (1), we can express the relations defining the constrained subspace, 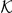, *parametrically* by
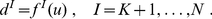
(3)where the functions 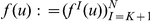 are the ones whose existence the Implicit Function Theorem guarantees. The coordinates 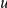 are thus termed *unconstrained* and they parameterize 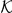, whereas the coordinates 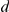 are called *constrained* and their value is determined at each point of 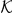 according to (3). In general, the functions 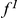 will depend on 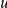, and the constraints will be said to be *flexible* [11]. In the particular case in which all the functions 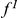 are constant along 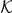, the constraints are called *hard*, and all the calculations are considerably simplified. In this work, we tackle the general, more involved, flexible case.

Of course, even if 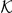 is regular in all of its points, the particular coordinates 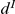 that can be solved need not be the same along the whole space. One of the simplest examples of this being the circle in 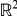, which is given by 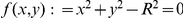, an implicit expression whose gradient is non-zero for all 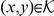. However, if we try to solve, say, for 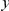 in the whole space 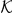, we will run into trouble at 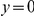; if we try to solve for 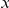, we will find it to be impossible at 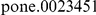. I.e., the Implicit Function Theorem does guarantee that we can solve for *some* of the original coordinates at each regular point of 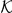, but sometimes the solved coordinate has to be 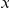 and sometimes it has to be 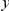. Nevertheless, we will assume this to be the case throughout this work, as is normally done in the literature [12]–[15], and thus we will consider that 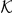 is parameterized by the *same* subset of coordinates 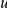 for all of its points.

It is also worth mentioning at this point that, not only from the physical point of view all the constraints dealt with in this work are just holonomic constraints, but also the wording used to refer to the two *flexible* and *hard* sub-types is multiple in the literature. The first sub-type is called *flexible* in refs. [13]–[16], *elastic* in [17], and *soft* in [15]; whereas the second sub-type is called *hard* in refs. [15], [16], just *constrained* in [13], or *holonomic* in [17], *rigid* in [14], [15], and *fully constrained* in [15]. Some of these terms are clearly misleading (*elastic*, *holonomic* or *fully constrained*), and, in any case, so many names for such simple concepts is detrimental to understanding in the field.

The situation is further complicated by the fact that, when studying the statistical mechanics of constrained systems, one can think about two different models for calculating the equilibrium probability density, whose names often collide with the ones used for defining the type of constraints applied. On the one hand, one can implement the constraints by the use of very steep potentials around the constrained subspace; a model sometimes called *flexible* [18], [19], sometimes called *stiff* [12], [20]. On the other hand, one can assume the D’Alembert principle [21] and hypothesize that the forces are just the ones needed for the system to never leave the constrained subspace during its dynamical evolution; a model normally called *rigid* [12], [18], [19]. The two statistical mechanics models have long been recognized to present different equilibrium probability distributions [18]–[20], [22], and this is the major concern in the literature when discussing them. In refs. [11], [12], the reader can find a very detailed discussion of this issue, which we only touch here briefly for completeness.

It is worth remarking that the two types of constraints and the two types of statistical mechanics models can be independently combined; one can have either the *stiff* or the *rigid* model, with either *flexible* or *hard* constraints, hence making any interference between the two sets of words undesirable. The wording chosen is this work is, on the one hand, fairly common, and on the other hand, non-misleading.

Now, if we take any physical observable 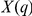, depending only on the coordinates (not on the momenta), and originally defined on the whole space, 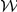, its *restriction* to 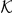 is given by
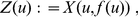
(4)where the symbol has been deliberately changed in order to indicate that 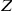 and 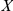 are different functions.

The derivatives of this observable along 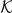 are thus
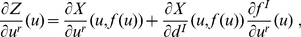
(5)where we have assumed the convention that repeated indices (like 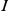 above) indicate a sum over the relevant range, and we have omitted (as we will often do) the range of variation of the index 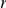.

In the case of hard constraints, i.e., when the functions 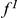 are all constant numbers 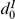, the above expression reduces to
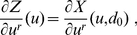
(6)where 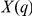 must be a known function of 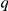 (in order to have a well-defined problem), and its derivative is typically easy to compute. However, if the constraints are of the more general, flexible form (the ones tackled in this work), the calculation of the partial derivatives 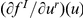 cannot be avoided.

If the constraints are assumed to be flexible, it is common in the literature of molecular modeling to define these functions 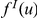 as the values taken by the coordinates 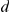 if we minimize either the total or the potential energy with respect to all 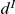 at fixed 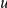 [11]–[15]. Since the energy functions used in molecular simulation are typically rather complicated, such as the ones in classical force fields, with a large number of distinct functional terms [23]–[28], or the effective nuclear potential arising from the solution of the electronic Schrödinger equation in the ground-state Born-Oppenheimer approximation [12], [29], the minimization of the energy with respect to the coordinates 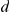 has to be performed numerically. Hence, the functions 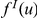, which are the output of this process, do not have a compact analytical expression that can be easily differentiated to include it in eq. (5) (this is even the case in very simple toy systems; see the Results and Discussion section).

In this work, we present a parameter-free, exact algorithm (up to machine precision) to calculate the derivatives 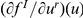 in such a case. Although several methods exist in the literature [13]–[15] for performing MD simulations with flexible constraints, nobody has dealt, as far as we are aware, with the computation of these derivatives. Since the general idea can be applied to any situation in which (1) we have flexible constraints, (2) that are defined in terms of the minimization of some quantity with respect to the constrained coordinates, we first introduce, the essential part of the algorithm based on these two points. Then, we develop a more sophisticated application of this idea to the calculation of the derivatives along the constrained subspace of the Euclidean coordinates of molecular systems; a problem that we faced in our group when trying to calculate the correcting terms associated to mass-metric tensor determinants that appear in the equilibrium probability density when constraints are imposed [11], [12], [30]. Finally, we perform a comparison between the results obtained with our exact algorithm and the calculation of the derivatives by finite differences; this serves the double purpose of numerically validating the algorithm and showing the limitations of the latter method, which needs the tuning of a parameter for each particular problem.

## Methods

### General Algorithm

As we mentioned in the Introduction, we assume that we are dealing with a constrained problem in which the functions 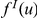 in eq. (3) are defined as taking the values of the constrained coordinates 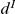 that minimize a given function, 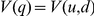, for each fixed 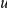, i.e.,
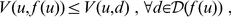
(7)where 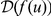 is a suitable open set in 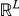 containing the point 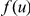. Depending on the particular application, one can ask the minimum that defines the functions 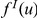 to be global or just local. However, in the cases in which 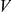 is the total or the potential energy of a complex molecular system, it may become very difficult to find its global minimum (due to the shear number of dimensions of the search space), and the local choice is the only reasonable one [11].

In order to calculate the derivatives along 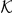 of any physical observable function of the coordinates 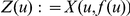, like the one defined in (4), we can always follow the finite-differences approach. However, as we discuss in the Results and Discussion section, finite differences presents intrinsic inaccuracies which are difficult to overcome, specially as the system grows larger. Let us now introduce a different way to calculate 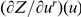 which does not suffer from this drawback.

The starting point is eq. (5) in the Introduction, which we copy here for the comfort of the reader:
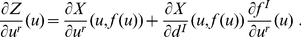
(8)


As we mentioned, the expression of 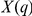, as well as the functions 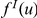, must be known if we wish to have a well-defined constrained problem to begin with. Therefore, the only objects that remain to be computed are the partial derivatives 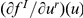.

If we assume that we have available some method to check that the order of the stationary point is the appropriate one (i.e., that it is a minimum, and not a maximum or a saddle point), we can write a set of equations which are equivalent to eq. (7), and which (implicitly) define the functions 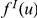:
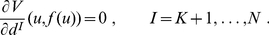
(9)


Now, we can take the derivative of this expression with respect to a given unconstrained coordinate 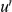:
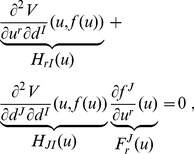
(10)where 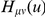, with 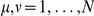, is the Hessian matrix of 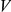 evaluated at 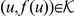, and 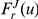 is the matrix of unknowns that we want to solve for. In the whole document, we adhere to the practice of using different types of indices in order to indicate different ranges of variation. Here, for example, 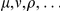 run from 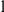 to 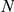; 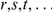 run from 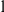 to 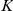; and 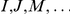 run from 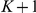 to 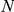. In the next section, we need to use more types of indices, but the idea is the same.

It is worth mentioning that similar equations to the ones above can be found in classical mechanics anytime that local coordinates are used (the coordinates 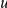 in this work). For example, the force in such a case is defined as 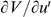 and the chain rule can be used in a similar way to what we do here. Note, however, that eq. (9) does not contain derivatives with respect to 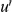, but to the constrained coordinate 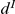. This makes the approach slightly different and, indeed, eq. (10) would become trivial in the most common hard situation tackled in the literature, where 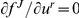, 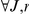.

Since we are, by hypothesis, in a minimum of 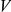 with respect to the constrained coordinates 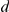, the constrained sub-block 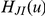 of the Hessian is a positive definite matrix, and therefore invertible. Hence, if we multiply eq. (10) by its inverse, denoted by 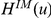, sum over 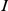, exploit the fact that 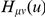 and 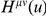 are symmetric, and conveniently rename the indices, we arrive at:
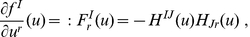
(11)which, as promised, allows us to find the exact derivatives 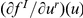 with the only knowledge of the Hessian of 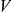 at the point 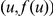, and, upon introduction of the result in eq. (8), also the derivatives along the constrained subspace 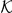 of any physical observable 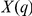.

As mentioned, several methods exist in the literature [13]–[15] to perform MD simulations with flexible constraints, however, none of them has tackled the calculation of these derivatives, which are very basic objects presumably to be needed in many future applications (see, e.g., refs. [11], [12]). Of course, it is always possible to compute derivatives using the simple and straightforward method of finite differences. In this work, we use finite differences as a way of validating the new, exact method and ensuring it is error free.

The accuracy of the new algorithm is only limited by the accuracy with which we can calculate the Hessian of 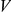 at 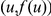 and invert it; there is no tunable parameter that we need to adjust for optimal accuracy, as in the case of finite differences (see below and also Results and Discussion). This makes a difference because, in classical force fields [26] and even in some quantum chemical methods (e.g., see chap. 10 of [31]), the Hessian can be calculated analytically, without the need of finite differences.

Although no optimization of the numerical cost has been pursued in this work, some remarks can be made about it, in comparison with the cost of the finite-differences approach. In order to calculate the partial derivatives 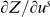 with respect to the unconstrained coordinates 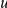 using finite differences, we need to:

1. Minimize 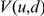 at fixed 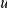 to find 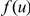 (this step is common with the new method introduced here).
2. Calculate 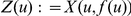 (this step is common with the new method introduced here).
3. Choose a displacement 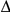 and minimize 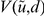 at the point 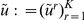, where 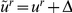 if 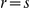 and 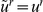 if 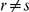. This yields 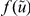 at a nearby point in 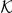 with 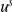 displaced a quantity 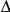 and the rest of unconstrained coordinates kept the same.
4. Calculate 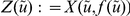.
5. Calculate

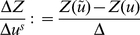
(12)as the finite-difference approximation to the sought derivative 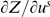 at the point 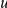.

Note that the third point of this finite-differences approach is essentially a linear stability analysis. When strongly non-equilibrium points are present, such as in the examples discussed in the last section, this approximation fails and the fact that the new algorithm introduced in this work uses only quantities defined *at the point* 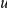 becomes even more important.

Now, assuming that we have a good enough guess for the parameter 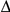, the cost of this procedure is dominated by the need to perform 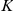 minimizations of the function 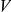, one in each of the directions corresponding to the unconstrained coordinates 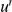. If we denote by 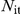 the average number of iterations needed for these minimizations to converge, and we define 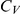 and 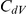 as the numerical costs (in computer time) of computing 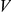 and its first derivatives with respect to the constrained coordinates 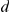, respectively, we have that the average cost of calculating the sought derivatives 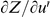 using finite differences will be 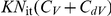 for local optimization methods such as the steepest descent or the conjugate gradient, or 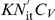 for Monte Carlo-based methods in which the derivatives of 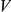 are not needed, such as simulated annealing [32].

On the other hand, the new algorithm does not require the extra minimizations, but it does require the calculation of the Hessian of 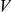 with respect to the internal coordinates (whose cost we call 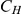), and the computation of the inverse of its constrained sub-block, 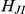, applied to each one of the 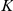 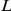-vectors 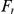 in eqs. (10) and (11), of cost 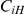; resulting in a total cost of 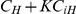.

The comparison between the two costs is not trivial and some remarks about it must be made: First, one must notice that the different individual costs involved, 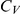, 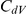, 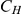 and 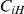, are strongly dependent on the characteristics (1) of the coordinates 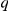 used and (2) of the function 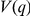. For example, if the coordinates 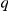 are the Euclidean ones and the function 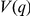 is the potential energy of a molecular system as modeled by a typical force field [23]–[28], the most direct algorithms for calculating 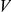 and its derivatives yield costs 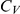, 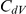 and 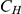 which are of order 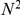, 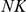 and 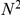, respectively [3]. However, if more advanced long-range techniques are used, such as the particle-particle particle-mesh (PPPM) method [33], the fast multipole method [34] or the particle-mesh Ewald summation [35], these costs can be reduced to order 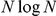 or even 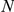 (for large 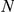 and forgetting prefactors). Also, as mentioned, if the coordinates used are not the Euclidean ones but some internal coordinates such as the ones used in this work, these costs must change in order to account for the transformation between the two. If force fields are not used but, instead, 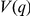 is the ground-state Born-Oppenheimer energy as calculated using Hartree-Fock [29], then the most naive implementations yield costs for 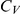, 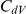 and 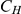 which are of order 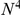 [31]. The cost, 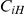, of calculating the inverse of 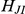 applied to a vector 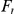 can range from order 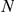 to order 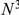 depending on the sparsity of the matrix [32], which, in turn, depends again on the coordinates used and on the structure of 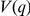. Finally, additional qualifications may complicate the comparison, such as the architecture of the computers in which the algorithms are implemented, parallelization issues, or the fact that, e.g., if we need the Hessian for a different purpose in our simulation, such as the calculation of the corresponding correcting term that appears both in the constrained stiff model and in the Fixman potential [12], then the ‘only’ computational step we are adding is the inversion of a matrix.

Despite the complexity and problem-dependence of the cost assessment, it must be stressed that, even in the cases in which the new algorithm turns out to be more expensive than the alternatives, the fact that it is exact and parameter-free might still make it the preferred choice in problems where high accuracy is needed. Although a parameter-free structure does not guarantee higher accuracy, in this case it does, since our method can be identified as the proper limit of the finite-differences scheme when 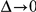. This is illustrated in Results and Discussion.

It is also worth remarking that the new method, as mentioned, is not needed to perform MD simulations, which can be run without calculating any of the derivatives tackled in this work [13]–[15]. Our method is only needed when some observable in which these derivatives are included (such as the aforementioned mass-metric tensor determinants) needs to be computed. In such cases, the only two options to get to the final result are either finite differences or our method, and the most convenient of the two has to be chosen; even if its cost is a burden.

### Application to Euclidean Coordinates of Molecules

In this section, we will apply the general algorithm introduced above to calculate the derivatives along the constrained subspace of the Euclidean coordinates of molecular systems in a frame of reference (FoR) fixed in the molecule. This problem has been faced by our group when trying to calculate the correcting terms associated with mass-metric tensor determinants that appear in the equilibrium probability density when flexible constraints are imposed [11], [12], [30]. More specifically, these derivatives are needed to calculate the determinant of the induced mass-metric tensor 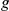 that appears in the constrained rigid model, according to the formulae derived in ref. [30].

In such a case, the system of interest is a set of 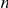 mass points termed *atoms*. The three Euclidean coordinates of atom 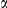 in a FoR fixed in the laboratory are denoted by 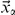, and its mass by 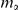, with 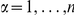. However, when no explicit mention to the atom index needs to be made, we will use 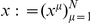 to denote the 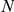-tuple of all the 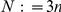 Euclidean coordinates of the system. The masses 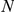-tuple, 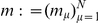, in such a case, is formed by consecutive groups of three identical masses, corresponding to each of the atoms.

Apart from the Euclidean coordinates, one can also use a given set of *curvilinear coordinates* (also called sometimes *general* or *generalized*), denoted by 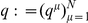, to describe the system. Both the coordinates 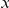 and 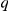 parameterize the whole space 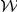, and the transformation between the two sets and its inverse are respectively given by
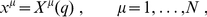
(13a)

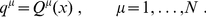
(13b)


We will additionally assume that, for the points of interest, this is a *proper* change of coordinates, i.e., that the *Jacobian matrix*

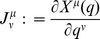
(14)has non-zero determinant.

**Figure 1:**
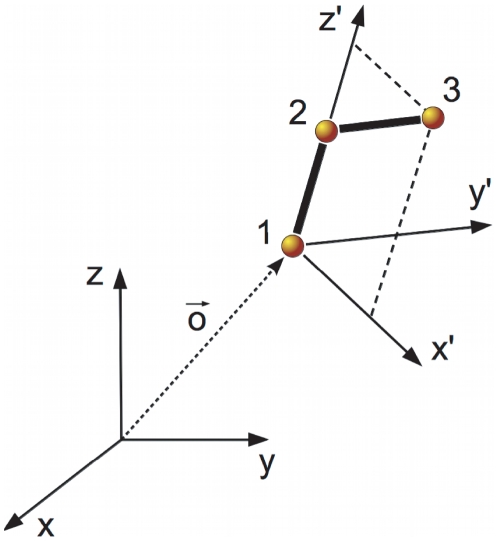
Definition of the frame of reference fixed in the system.

Now, we define a particular FoR *fixed in the system* to perform some of the calculations. To this end, we select three atoms (denoted by 1, 2 and 3) in such a way that 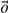, the position in the FoR of the laboratory of the origin of the FoR fixed in the system, is the Euclidean position of atom 1 (i.e., 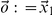). The orientation of the FoR 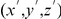 fixed in the system is chosen such that atom 2 lies in the positive half of the 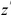-axis, and atom 3 is contained in the 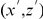-plane, with projection on the positive half of the 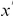-axis (see fig. 1). The position of any given atom 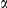 in the new FoR fixed in the system is denoted by 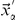. Also, let 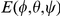 be the Euler rotation matrix (in the ZYZ convention) that takes a free 3-vector of primed components, 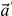, to the FoR fixed in the laboratory, i.e., 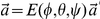 [21].

Although the aforementioned curvilinear coordinates 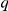 are a priori general, it is very common to take into account the fact that the typical potential energy functions of molecular systems in absence of external fields do not depend on 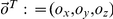 nor on the angles 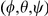, and to consequently choose a set of curvilinear coordinates split into 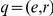, where the first six are these *external coordinates*, 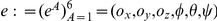. As we mentioned before, 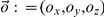 describes the overall position of the system with respect to the FoR fixed in the laboratory, and its overall orientation is specified by the angles 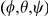. The remaining 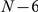 coordinates 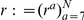 are called *internal coordinates* and determine the positions of the atoms in the FoR fixed in the system [36], [37]. They parameterize what we shall call the *internal subspace* or *conformational space*, denoted by 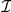, and the coordinates 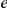 parameterize the *external subspace*, denoted by 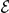; consequently splitting the whole space as 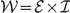 (denoting by 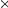 the Cartesian product of sets).

The position, 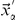, of any given atom 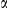 in the axes fixed in the system is a function, 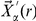, of only the internal coordinates, 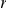, and the transformation from the Euclidean coordinates 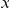 to the curvilinear coordinates 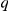 in (13) may be written more explicitly as follows:
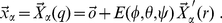
(15)


Although general constraints affecting all the coordinates 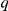 [like those in (1)] can be imposed on the system, the already mentioned property of invariance of the potential energy function under changes of the external coordinates, 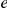, together with the fact that the potential energy can be regarded as ‘producing’ the constraints [12], make physically frequent the use of constraints involving only the internal coordinates, 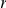:
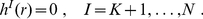
(16)


Under the common assumptions in the Introduction, these constraints allow us to split the internal coordinates as 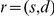, where the first 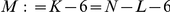 ones, 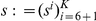, are called *unconstrained internal coordinates* and parameterize the *internal constrained subspace*, denoted by 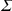. The last 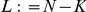 ones, 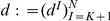, correspond to the *constrained coordinates* in the Introduction and are called. The external coordinates, 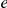, together with the unconstrained internal coordinates, 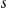, constitute the set of all *unconstrained coordinates* of the system, 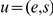, which parameterize the constrained subspace 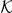, being 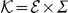.

In this situation, the constraints in eq. (16) are equivalent to
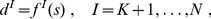
(17)and the functions 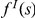 are defined as taking the values of the coordinates 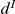 that minimize the potential energy with respect to all 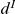 at fixed 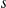 [11], [12], [15].

Finally, if these constraints are used, together with (16), the Euclidean position of any atom in the constrained case may be parameterized with the set of all unconstrained coordinates, 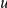, as follows:
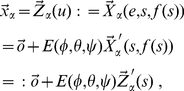
(18)where the name of the transformation functions has been changed from 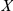 to 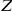, and from 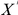 to 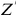, in order to emphasize that the dependence on the coordinates is different between the two cases.

In order to calculate the derivatives along 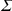 of the primed atoms positions, 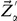, with respect to the unconstrained internal coordinates 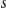 (needed, for example, in eq. (28) of ref. [30] to compute the determinant of the induced mass-metric tensor 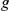), we first differentiate with respect to 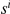 in 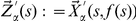, arriving to the analogue to eq. (8):
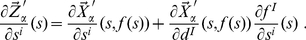
(19)


Now, the derivatives 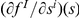 can be calculated using the general algorithm introduced in the previous section simply noticing that, in this case, 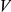 is precisely the potential energy of the system. Therefore, the only objects that remain to be computed are the derivatives 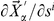 and 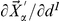, which can be known analytically (they are *geometrical* [or *kinematical*] objects, i.e., they do not depend of the potential energy). We now turn to the derivation of an explicit algorithm for finding them and thus completing the calculation that is the objective of this section.

In the supplementary material of ref. [30], we give a detailed and explicit way for expressing any ‘primed’ vector 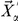 as a function of all the internal coordinates, in the particular coordination scheme known as SASMIC [37]. We could take the final expression there [eq. (5)] and explicitly perform the partial derivatives, however, we shall follow a different approach that is both more straightforward and applicable to a larger family of Z-matrix-like schemes for defining internal coordinates.

In non-redundant internal coordinates schemes, whether they are defined as in ref. [37] or not, each atom is commonly regarded as being incrementally added to the growing molecule for its coordination. This means that the position of the 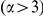-th atom in the body-fixed axes is uniquely specified by the values of three internal coordinates that are defined with respect to the positions of three other atoms with indices 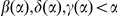. This is a very convenient practice, and we will assume that we are dealing with a scheme that adheres to it.

Normally, the first of the three internal coordinates used to position atom 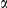 is the length of the vector joining 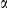 and 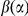. Atom 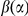 is commonly chosen to be covalently attached to 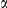 and, then, the length of this vector is naturally termed *bond length*, and denoted by 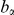. A given function 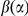 embodies the protocol used for defining this atom, 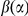, to which each ‘new’ atom 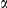 is (mathematically) attached; a superindex, as in 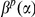, indicates composition of functions, and the iteration of such compositions allows us to trace a single-branched chain of atoms that takes from atom 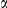 to atom 1, at the origin of the ‘primed’ axes. If 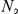 is a number such that 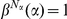, this chain is given by the following set:
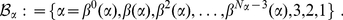
(20)


It is clear that, if we now change a given bond length 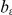 associated to atom 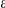, atom 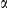 will move if 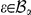; simply because atom 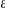 *will* move and 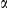 has been positioned in reference to atom 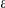
’s position. Thus, if we define
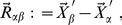
(21)for any 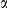, 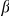, and accordingly denote by 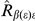 the unitary vector in the ‘primed’ FoR that points from atom 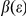 to atom 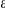, a change in the bond length associated to 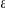 from 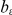 to 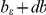 (while keeping the rest of the internal coordinates constant) will translate all atoms 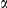 such that 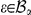 a distance 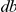 along 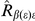, having
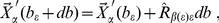
(22)and hence
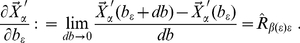
(23)


**Figure 2:**
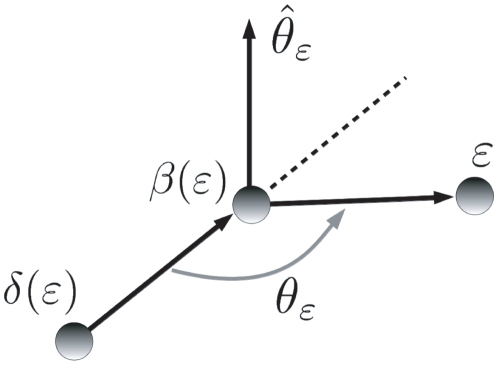
Rotation associated to a change in a bond angle. Definition of the *bond angle* 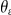, associated to atom 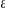, and the unitary vector 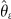 corresponding to the direction around which all atoms 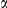 with chains 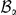 containing 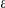 rotate if 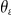 is varied while the rest of internal coordinates are kept constant.

The second internal coordinate, after 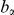, that is typically defined to position atom 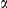 with respect to the ‘already positioned’ part of the molecule is a so-called *bond angle* 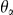. To define this angle, we need an additional atom associated with 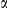, which we could denote by 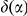. Although one can in principle think of the possibility of using different atoms 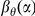 and 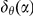 to define the bond angle than the one used to define the bond length, the common practice in the literature is to use the same three atoms 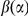, 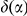 and 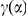, to define the three internal coordinates associated to 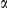. This is also the choice in the SASMIC scheme and the one in this work. The angle 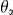 is thus defined as 180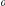 minus the angle formed between the vectors 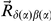 and 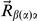 (see fig. 2).

Now, the reasoning is the same as in the case of the derivative with respect to 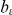: For every atom 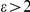 that is the ‘tip’ of the bond angle 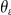, the changes in this angle (keeping the rest of internal coordinates constant) will move atom 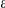 and therefore all atoms 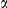 that contain atom 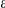 in the chain 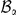 that links them to atom 1.

If we now look at fig. 2, we see that a change from 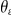 to 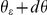 amounts to rotate all atoms 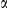 that contain 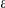 in their chain to the origin an angle 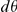 around the unitary vector 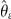, which is defined by
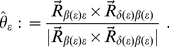
(24)


The result, 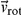, of rotating a vector 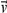 around the direction given by the unitary vector 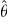 an amount 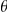 is given by the well-known *Rodrigues’ rotation formula* [21], [38]:
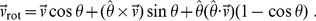
(25)


However, notice that, in order to define a rotation, it is not enough to specify the angle 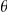 and the rotation axis 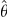, but one additionally needs to specify a fixed point (which can actually be any of the points in a fixed line in the direction of 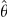). Therefore, the above expression is only correct for either ‘free’ vectors 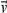 (i.e., those that are not associated to a given point in space), or for vectors 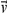 whose starting point lies in the aforementioned fixed line.

The fixed point for the rotation we are interested in can be chosen to be 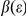 and, using eq. (25), we have that
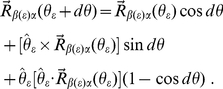
(26)


Then, keeping the terms up to first order in 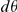, we can easily compute the derivative:
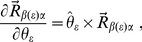
(27)which, since a variation of 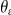 does not move atom 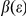 (i.e. 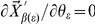), allows us to conclude that
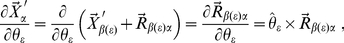
(28)if 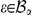.

**Figure 3:**
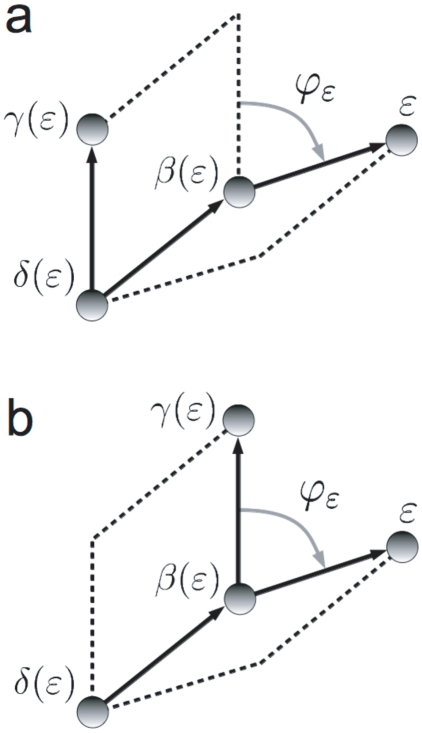
Rotation associated to a change in a dihedral angle. Definition of the *dihedral angle* 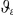, associated to atom 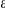. The positive sense of rotation is indicated in the figure, and we can distinguish between two situations regarding covalent connectivity: **a**) *principal dihedral angle*, and **b**) *phase dihedral angle* (see ref. [37]).

The third and last internal coordinate that is usually defined to position atom 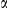 is a so-called *dihedral angle* 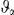. To define this angle, we need a third atom associated with 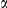, which we could denote by 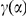. The angle 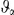 is thus defined as the oriented angle formed between the plane containing atoms 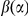, 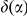 and 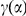 and the plane containing atoms 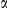, 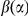 and 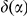. The positive sense of 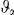 is the one indicated in fig. 3, and, although it is common to find two different covalent arrangements of the four atoms 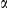, 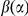, 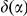 and 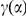, termed *principal* and *phase* dihedral angles, respectively [37], this does not affect the mathematical definition of 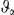 given in this paragraph, nor the subsequent calculations.

Regarding the derivative of the ‘primed’ position of atom 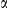 with respect to a given 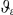, the only difference with the bond angle case is that, now, the rotation is performed around the direction given by the unitary vector 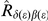 (see fig. 3). The fixed point can be again chosen as 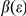, and eq. (27) (changing 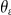 by 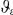 and 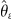 by 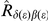), as well as the fact that changes in 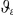 do not move atom 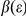, still hold. Therefore,
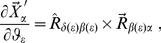
(29)if 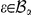.

In order to decide whether or not atom 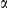 will move upon changes in internal coordinates associated to atoms 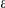 that *do not* belong to 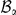 we must first finish the story about internal coordinates definition. Since the argument above to show that 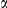 moved when 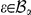 was that 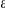 *itself* moved and it was used to position 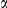, we must ask

1. whether or not there can be atoms that are also used to position 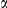 but that do not belong to 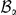, and
2. what happens when we change the internal coordinates associated to them.

**Figure 4:**
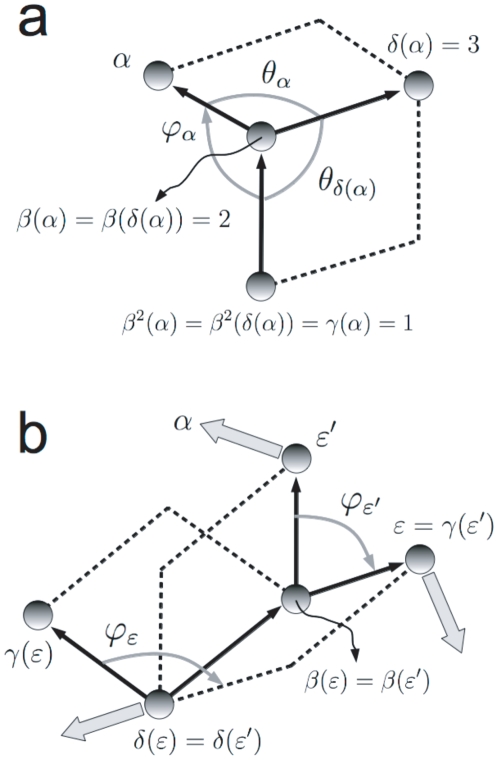
Special cases. Special cases of atoms that do not belong to the chain 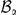 connecting 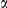 to atom 1, but that are nevertheless used to position 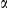.

The answers to these two questions depend on the particular scheme used to define the internal coordinates, and we will tackle them referring to the SASMIC scheme [37], which is the one used in this work: According to the SASMIC rules, there are only two situations in which an atom 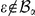 can be used to position atom 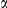, and they are depicted in fig. 4.

The first case, in fig. 4a, attains only the first atoms of the molecule. Typically, atom 1 is not a first-row atom, but a hydrogen (such is the case of the three molecules studied, for example, in Results and Discussion). Hence, after positioning atoms 2 and 3, which are typically first-row, it is more representative to choose atom 3 as 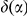 and atom 1 as 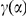 when positioning the rest of the atoms 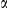 attached to atom 2. This makes 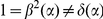 and hence 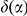 qualifies as an atom that is used to position 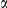 but which is not included in the chain 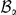.

The second case, in fig. 4b, corresponds to the situation in which the molecule divides in two branches, and it can happen all along its chemical structure. If atom 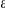 is the atom that defines the only principal dihedral over the bond connecting 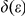 and 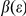 (in the SASMIC scheme, only one principal dihedral can be defined on a given bond [37]), and atom 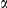 belongs to a different branch than the one beginning in 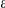 (the branches are indicated with grey broad arrows), then the starting atom 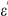 of the branch to which 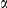 belongs (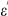 can be 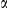 itself) must be positioned using a phase dihedral in which 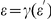. Thus, 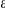 is an atom that is used to position 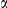, but which does not belong to the chain 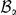 connecting 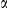 to atom 1.

In principle, any change in the internal coordinates of atom 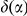, in the first case, or in those of atom 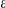, in the second case, may move atom 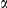, however, due to the geometrical characteristics of the internal coordinates, this is not the case.

For example, it is easy to see that, in the case depicted in fig. 4a, a variation of the bond length 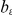 (denoting 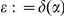) does not move atom 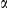. Regarding the angles, the dihedral 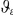 is not defined because 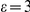, and a change in 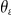 can be seen to rotate atom 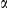 with fixed point 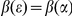 and around the axis given exactly by 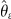 as defined in eq. (24). (It is not trivial to see that this motion keeps all the rest of internal coordinates constant, specially the phase dihedral 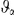. The authors found it helpful to imagine that atoms 1, 2 and 3 lie in the plane of the paper, with 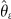 and 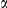 coming out of it towards the reader; the first orthogonally and the second not.) Therefore, the derivative of the Euclidean position of atom 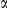 with respect to 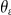 is also given by eq. (28) in this special case.

In the situation shown in fig. 4b, one can see that neither a change in 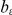 nor in 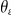 move atom 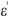 nor 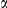. However, if we change 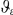, we need to move atom 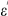 if we want to keep 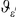 constant. Therefore, atom 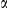 moves in such a case and it does so by rotating with the same fixed point 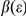 and the same axis 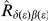 as in the simpler cases depicted in fig. 3. This means that, again, we can calculate the sought derivative using the already justified eq. (29).

In summary, only changes in bond lengths associated to atoms 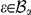 affect the position of atom 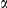:
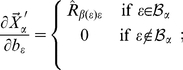
(30)changes both in bond angles associated to atoms 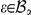 and to 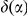 in fig. 4a affect the position of atom 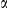:
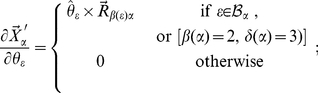
(31)and changes both in dihedral angles associated to atoms 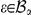 and to those that define the principal dihedral at a branching point that leads to atom 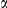 (see fig. 4b) can affect the position of atom 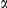:
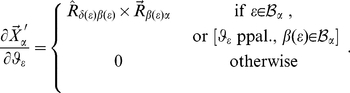
(32)


Finally, the outline of the algorithm for calculating the sought derivatives 
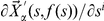
 along the constrained subspace 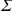 is:

1. Calculate the chain 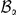 that connects atom 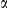 with atom 1 and identify the special cases depicted in fig. 4.
2. Calculate the derivatives 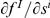 by solving the system of linear equations in (11).
3. Calculate the geometric derivatives 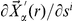 and 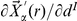, for 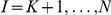, using eqs. (30), (31) and (32).
4. Plug all the calculated quantities into eq. (19) et voilà.

## Results and Discussion

In this section, we compare the finite-differences approach (see Methods) to the new algorithm introduced in this work with two objectives in mind: the validation of the new scheme, and the identification of the most important pitfalls of the finite-differences technique, which are absent in the new method. It is worth stressing again that the method presented here is the first of its kind, as far as we are aware, and the finite-differences scheme is just a very natural and straightforward method that is always available when derivatives need to be calculated. In fact, the pitfalls of finite differences which we highlight in this section are very well known, although they have been seldomly presented in the context of molecular force fields. We hope that this section can be additionally useful to revisit this classical topic from a new angle.

**Figure 5:**
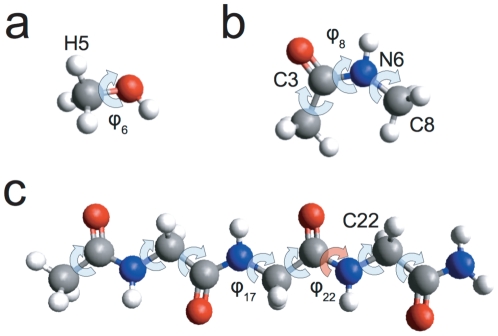
Molecules used in the numerical calculations in this section. (**a**) Methanol, (**b**) N-methyl-acetamide (abbreviated NMA), and (**c**) the tripeptide N-acetyl-glycyl-glycyl-glycyl-amide (abbreviated GLY3). Hydrogens are conventionally white, carbons are grey, nitrogens blue and oxygens red. The unconstrained dihedral angles that span the corresponding spaces 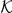 are indicated with light-blue arrows, and some internal coordinates and some atoms that appear in the discussion are specifically labeled. The constrained dihedral angle 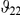 is indicated by a red arrow in GLY3.

To these two ends, we have applied the more specific algorithm introduced in the previous section for the calculation of the derivatives of the Euclidean coordinates of molecular systems to the three biological species in fig. 5: methanol, N-methyl-acetamide (abbreviated NMA), and the tripeptide N-acetyl-glycyl-glycyl-glycyl-amide (abbreviated GLY3). For each one of these molecules, a number of dihedral angles describing rotations around single bonds (and indicated with light-blue arrows in fig. 5) have been chosen as the unconstrained internal coordinates, 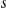, spanning the corresponding constrained internal subspace 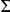. The rest of internal coordinates 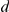 (bond lengths, bond angles, phase dihedrals, and principal dihedrals over non-single bonds) are flexibly constrained as described in the previous sections. The numeration of the atoms and the definition of the internal coordinates follow the SASMIC scheme, which is specially adapted to deal with constrained molecular systems [37].

For methanol and NMA, due to the small dimensionality of their constrained subspaces, the working sets of conformations have been generated by systematically scanning their unconstrained internal coordinates at finite steps. For methanol, we produced 19 conformations, in which the central dihedral, 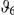, ranges from 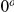 to 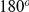 in steps of 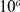. Similarly, the systematic scanning of the unconstrained dihedrals in NMA produced a set of 588 conformations in which the first and last angles, 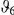 and 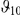, range from 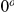 to 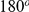, and the central one, 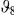, ranges from 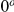 to 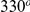, all in steps of 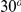. For GLY3, and in view of the dimensionality of its constrained subspace, 1368 conformations were generated through a Monte Carlo with minimization procedure.

At each one of these conformations, defined by the value of the unconstrained internal coordinates 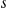, the constrained coordinates 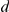 were found by minimizing the potential energy 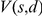 at fixed 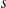, thus enforcing the constraints 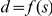 described in Methods. Let us remark that this fixing of the coordinates 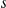 is just an algorithmic way of sampling the constrained subspace defined by the relations 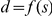, and it does not imply that the coordinates 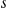 are constrained; indeed, they could take any value in the set of conformations, whereas the constrained coordinates 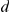 are fixed by the aforementioned relations. The potential energy and force-field parameters were taken from the AMBER 96 parameterization [39], [40], and local energy minimization with respect to the constrained coordinates was performed with Gaussian 03 [41]. At the minimized points, the Euclidean coordinates, 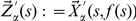, of all atoms in the system-fixed axes defined in the Methods section were also computed.

In order to find the partial derivatives 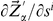 at the generated points by finite differences, we produced, for each conformation in the working sets, 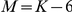 additional conformations, each one with a single coordinate 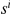 displaced to 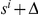. After the re-minimization of the constrained coordinates at the new points, we were in possession of all the data needed to compute the estimate of the sought derivative in eq. (12) for all unconstrained coordinates. In order to assess the behaviour and accuracy of the finite-differences approach, we performed these calculations for the values 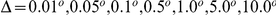.

On the other hand, to calculate the derivatives 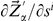 using the new scheme introduced in Methods, we do not need to perform any additional minimization, but we need to know the Hessian matrix of the second derivatives of 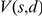 with respect to the internal coordinates. The Hessian in internal coordinates was calculated with the Gaussian 03 package [41].

**Figure 6:**
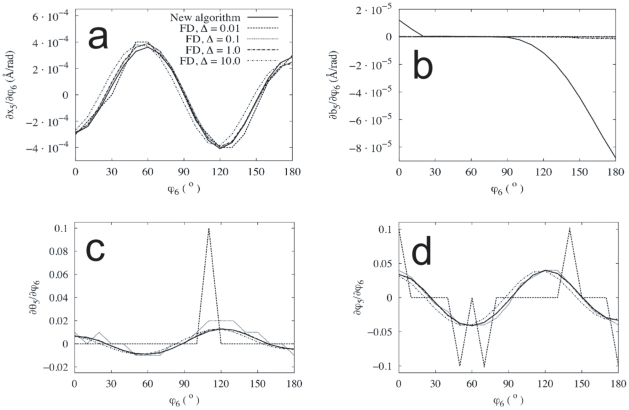
Derivatives of some selected coordinates of methanol. Derivatives of (**a**) the 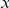 coordinate of atom 5 in methanol, (**b**) the bond length 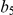 associated to it, (**c**) the bond angle 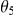, and (**d**) the dihedral angle 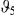 as a function of the unconstrained coordinate 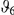. Both the results of the new algorithm and those obtained by finite differences (FD) are depicted. The key for the different types of line is the same in the four graphs.

In order to compare the two methods, we turn first to the smallest system: methanol. In fig. 6a, we can see the value of the derivative 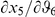 of the 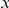-coordinate (in the ‘primed’ axes, but we drop the prime from now on) of hydrogen number 5 (see fig. 5) with respect to the unconstrained dihedral angle 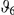 that describes the rotation of the alcohol group with respect to the methyl one. We can see that the agreement between the new algorithm and the finite-differences approach is good but not perfect, and that the discrepancy between the two is larger for the smallest (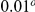) and largest (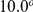) values of 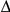 depicted in the graph.

To track the source of this difference, we can take a look at eq. (8), which gives the derivative 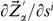 as a function of simple, ‘geometrical’ terms, 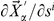 and 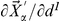, and the numerical derivatives 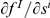. Of course, the choice of one method or another does not affect the former, but only the latter. In the particular case of 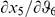 in fig. 6a, if we remove the terms that are zero according to the rules in eqs. (30), (31) and (32), eq. (8) becomes
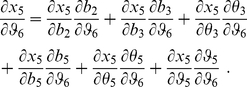
(33)


The numerical derivatives appearing in this expression that are related to the three constrained coordinates associated to atom 5 are shown in figs. 6b, 6c and 6d, respectively, where we can see that the discrepancy between the new algorithm and the finite-differences approach is more significant. For the bond angle 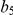 in fig. 6b, we see that the derivative predicted by finite differences is close to zero for all values of 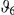 and for all the tested 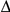
s, while the behaviour given by the new algorithm is more rich and substantially different. This large discrepancy is produced by the fact that bond lengths are very stiff coordinates in the energy function that we have used here, together with the default precision of the floating point numbers provided by Gaussian 03 outputs. In table 1, we can see indeed that the last significant figure of bond length 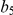 only starts to change for 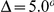, which makes any algorithm based on finite differences very unreliable for this particular quantity if small values of 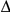 are used. The bond angles and dihedral angles, on the other hand, are somewhat less stiff than bond lengths, as it can also be seen in tab. 1. This makes their derivatives by finite differences more reliable, as one can observe in figs. 6c and 6d, where the discrepancy with the new method is apparent for small 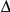, but becomes gradually smaller as we increase it. Of course, since, in the new method presented in this work, all quantities are computed at the non-displaced point 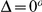, the problem regarding the number of significant figures does not appear. It is also worth remarking that, in the case of finite differences, the point in which this issue will appear depends on the number of bits used to represent coordinates, but it will always appear for some small enough value of 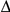.

**Table 1:** Stiffness of the constrained coordinates in methanol.

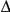 ( 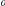 )	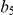 (Å)	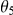 ( 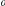 )	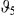 ( 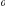 )
0.0	1.090694	109.403	119.296
0.01	1.090694	109.404	119.296
0.05	1.090694	109.404	119.297
0.1	1.090694	109.405	119.299
0.5	1.090694	109.409	119.312
1.0	1.090694	109.415	119.329
5.0	1.090693	109.462	119.474
10.0	1.090692	109.525	119.671

Values of the constrained coordinates associated to atom 5 of methanol for different displacements 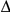 in the unconstrained coordinate 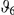. The values correspond to the conformation with 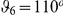, and the number of significant figures presented is the default one provided by Gaussian 03.

**Figure 7:**
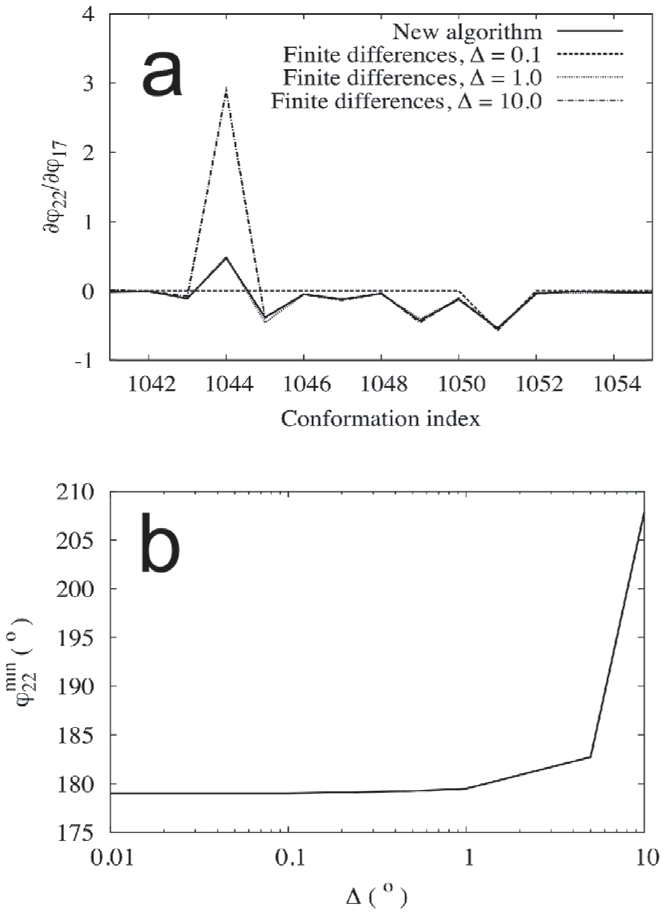
Metastability of the local minima in GLY4. (**a**) Derivative 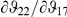 of the constrained dihedral angle 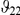, describing a peptide bond rotation in GLY3, with respect to the unconstrained coordinate 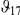 for a selected set of conformations in the working set. (**b**) Minimum-energy value of the constrained dihedral angle 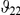 in the conformation 1044 of GLY3 for different values of the displacement 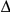 in the unconstrained coordinate 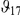.

As we noticed in fig. 6a, also in the case of the constrained internal coordinates the difference between the two methods starts to grow again when 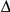 reaches 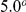 or 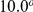. This is easily understood if we think that only in the 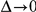 limit the estimate in eq. (12) converges to the actual value of the partial derivative. In fact, as the complexity of the system increases, the error introduced at large 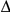 may come not only from continuous changes in the location of the constrained minima, but also, as fig. 7 suggests, it may occur that, at a certain value of 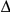, the very *identity* of the minima is altered, thus introducing potentially larger errors. In fig. 7a, we can see that the derivative 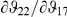 in GLY3 presents an unusually large error at the conformation 1044. In fig. 7b, we see that the minimum-energy value of 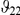, which is the dihedral angle associated to carbon 22, describing the rotation around a given peptide bond (see fig. 5c), presents an abrupt change when 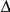 reaches 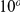. If we think that the energy landscape of GLY3 is indeed a complex and multidimensional one, it is not difficult to imagine that, as we change 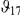, i.e., as we increase 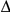, the energy landscape is so altered that some minima disappear, some other appear, and the energy ordering among them is changed. In such a case, the structures found by the minimization procedure will be rather different between, say, 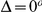 and 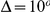, thus producing a large error in the derivatives calculated by finite differences. Again, the new algorithm, which only uses quantities calculated at 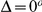, does not suffer from this drawback.

**Figure 8:**
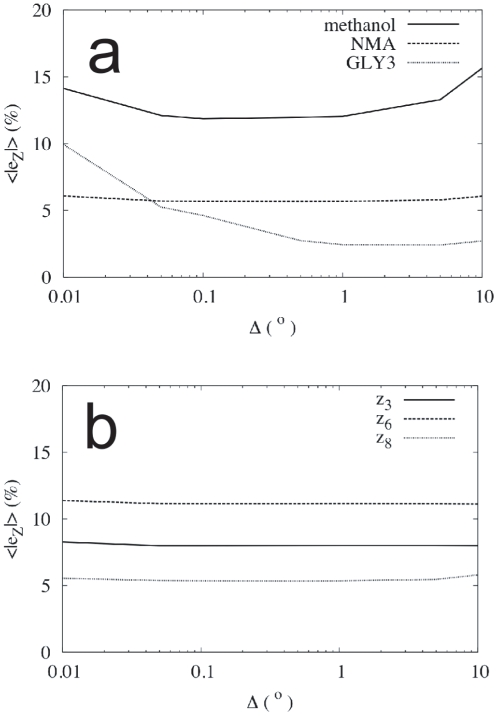
Dependence of the error as a function of 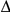. Average normalized error in the derivatives by finite differences as a function of 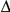 (see the text for a more precise definition). (**a**) Error averaged to all conformations and all atoms of the three molecular systems studied. (**b**) Error averaged to all conformations of the 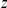-coordinate of three particular 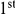-row atoms in NMA.

To sum up, the finite-differences method contains two sources of error which the new method does not present: one at small values of 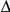, related to the finite precision of the floating point numbers representing the internal coordinates, and the other at larger values of 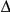, stemming from the very definition of the partial derivative by finite differences, and aggravated by the complexity of the energy landscapes of large systems. If the derivatives are to be calculated using finite differences, an optimal value of 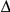 must be chosen in each case so that the possible error is minimized. However, already in the simple example of methanol, we saw that the derivatives of different observables, in the same system, may behave differently as we change 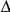 (compare the bond length derivative in fig. 6b with that of the angles in figs. 6c and 6d). In fig. 8, we additionally see that the search for the optimal 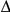 may be further complicated by the fact that the behaviour found also depends (strongly) on the system studied, and, in the case of the derivatives of the Euclidean coordinates, on the position of the atom in the molecule.

In fig. 8a, we have plotted the normalized average of the absolute value of the error in the derivatives of the Euclidean coordinates, 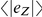, as a function of 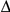 for the three molecular systems studied. This quantity is defined, for a given unconstrained coordinate 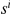, as
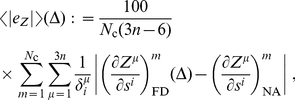
(34)where the index 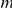 indicates the conformation in the working set, running from 1 to 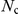, FD stands for ‘finite differences’, NA for ‘new algorithm’, and 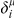 is a normalizing quantity for each coordinate 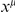 chosen as
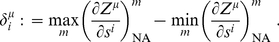
(35)


The graphics in fig. 8a of this quantity correspond to the unconstrained dihedral angles 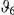, 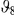 and 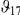 of methanol, NMA and GLY3, respectively (see fig. 5). We observe that the average error as a function of 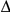 presents significantly different behaviours in the three molecules, never being smaller than a 2%. Additionally, in fig. 8b, we show the same error but this time individualized to the 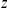-coordinate of three different 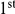-row atoms of NMA: C3, N6 and C8. Although the overall behaviour of the error is similar for the three atoms, its size is not.

All in all, we see that the need to tune for the optimal 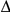 in the finite-differences approach not only produces unavoidable errors, but also it must be done in a per-system, per-observable basis, clearly complicating and limiting the use of this technique. The new algorithm, on the other hand, is only affected by the source of error related to the accuracy with which the Hessian matrix of the potential energy can be calculated and inverted; apart from this, which is a general drawback of any method implemented in a computer, its mathematical definition is ‘exact’, in the sense that it does not contain any tunable parameter, like 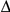, that must be adjusted for optimal accuracy in each particular problem.

Also, and more importantly (since the failure of finite differences was indeed predictable) the good coincidence between the newly introduced, somewhat more involved method and the straightforward finite-differences scheme for the smallest system and in some intermediate range of values of 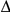 allows us to regard the new scheme as validated and error-free.

Finally, despite what we discussed in the Methods section, namely, that we have not pursued here the numerical optimization of the algorithm introduced, being our main interest to present the general theoretical concepts and to show that the new method is exact and reliable, we close this section with an example of a toy system to provide a clue that the new technique is at least feasible. Before introducing the toy system it is worth noting that the examples tackled in this section are just particular cases, but the technique can be used in different systems and with different potential energy functions. When looking at the computer costs presented below, the reader should bear in mind that they may be not very significant (due to the aforementioned lack of optimization) and not very relevant (due to the choice of a small toy system and a given potential energy function). Of course, if any production runs using the new algorithm are attempted, a thorough numerical optimization and assessment should be performed, which we deem to be a very important next step of our work.

**Figure 9:**
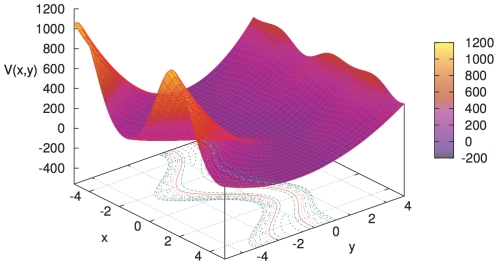
Potential energy of the toy system in eq. (36). The range of 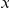 and 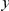 corresponds to the one explored in this work. Contour level lines and colour level indication in the surface have been added for visual comfort. All units are arbitrary.

The toy system is a 2-dimensional one, with positions 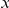 and 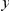, and the following potential energy (see fig. 9):
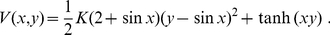
(36)


If we take a large enough 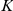, say 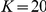, we see that the system will present a strong oscillatory motion in the 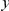 coordinate, around approximately 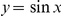 (but not exactly, since the term 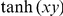 slightly modifies the position of the minimum), and with harmonic constant approximately equal to 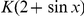. In the spirit of this work, since, due to energetic reasons, the value of 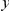 will seldomly move far away from the value that minimizes 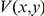 for each 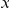, denoted by 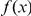 and implicitly defined by the following equation:
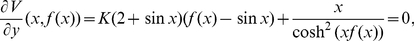
(37)we can kill this oscillatory motion by assuming that a flexible constraint 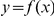 exists. In such a case, 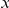 plays the role of the whole set of unconstrained coordinates 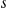 in the general formalism, and 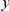 plays the role of the whole set of constrained coordinates 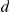.

Now, if we perform a ‘molecular dynamics’ of this system, then we may need at some point to compute the derivative with respect to 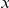 of some observable 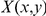 restricted to the constrained subspace 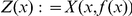 (for example, we may need this to calculate mass-metric tensor corrections at each time step [12]). We can do so by using finite differences or the new technique introduced in this work. As we discussed in the Methods section, for both approaches we will need to perform a minimization of 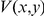 at each fixed 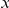 in order to find 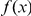 and 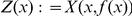, hence, being this step common, we will not consider it for the assessment of the differences in computational cost between the two methods. The additional computations that will decide which method is faster are:

- **For finite differences:** Choose a displaced value of the unconstrained coordinate 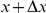, minimize 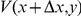 with respect to 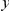 in order to find 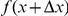, as well as 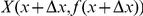, and finally calculate the finite-differences estimation of the sought derivative:

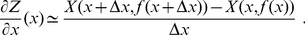
(38)

- **For the new method:** Calculate the objects in eq. (11), perform the required inversion to find 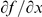, calculate the objects in eq. (5), and finally find 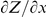 using this last expression. In this simple case, all the objects to be computed are:

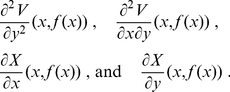
(39)The second-order derivatives of the potential energy can be easily calculated:
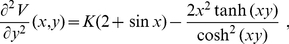
(40a)

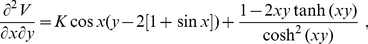
(40b)and we can use them to find the derivative of 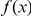 through eq. (11):
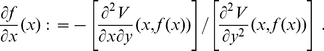
(41)


The particularization of eq. (5) to this simple case is
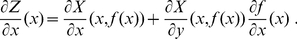
(42)


In this section, for illustrative purposes, we have chosen a simple observable 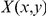:
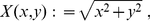
(43)i.e., the distance of the particle to the origin of coordinates. Hence, the remaining objects that we need to compute in order to apply the new technique to this problem are
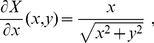
(44a)

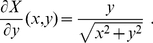
(44b)


We have calculated 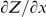 using both techniques for 11 different values of 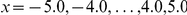. This calculation has been performed in a desktop iMac with a 2.66 GHz Intel Core 2 Duo processor and 4GB of 1067 MHz DD3 RAM memory, running MacOSX Snow Leopard. The same compilation-time optimizations have been used in the two cases, and the common times have been subtracted as indicated before. It is also worth remarking that we have used Brent’s method [32] for minimizing 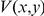, and a different choice will change the comparison. In these conditions, the new technique has proved approximately one order of magnitude faster than finite differences, elapsing 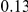 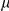
s/point vs. 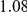 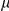
s/point.

In summary, in this work, we have introduced a new, exact, parameter-free method for computing the derivatives of physical observables in systems with flexible constraints. The new algorithm has been numerically validated in small molecules against its most natural alternative, finite differences. In doing so, numerous pitfalls of the latter method have been demonstrated, all arising from the fact that it contains a tunable parameter that has to be optimally adjusted in each particular problem at hand. In a number of numerical experiments, we have shown that the finite-differences approach contains two unavoidable sources of error that are not present in the new method: On the one hand, the finite number of significant figures used to represent, in computers, the values of the optimized coordinates, together with the fact that these constrained coordinates are typically very stiff, make the changes in this quantities often unobservable or at least badly resolved, thus rendering the finite-differences derivatives unreliable for small values of the displacement parameter 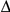. On the other hand, the very fact that finite-differences derivatives only converge to the true ones for 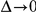, complicated with the possibility that the energy landscapes of complex molecular systems may significantly change their structure when the unconstrained coordinates are displaced, introduce new errors as 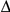 increases. These two sources of errors combined make compulsory the search of an optimal value of 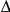 in each particular case, and also establish a minimum error below which is not possible to go, as it can be seen in fig. 8. Also, using a simple toy system, we have shown that the new technique can be faster than finite differences in certain situations. The new method introduced here, and it is already being successfully used in a number of works in progress in our group to compute the correcting terms appearing in the equilibrium probability distribution when flexible constraints are imposed on the system [11]. Moreover, given the almost ubiquitous occurrence of the concept of constraints all throughout the fields of computational physics and chemistry, it is expected that the method described in this work will find many applications in present and future problems. Some examples have been already mentioned in the introduction, notably the case of ground-state Born-Oppenheimer MD [42], [43] (using, e.g., Hartree-Fock [29]), which can be regarded as a flexibly constrained problem in which the soft coordinates are the nuclear positions 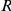, the hard ones are the electronic orbitals 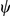, and the function to be minimized is the expected value 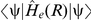 of the 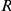-dependent electronic Hamiltonian in the 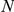-electron Slater determinant 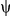.
