# Correction: An Exact Expression to Calculate the Derivatives of Position-Dependent Observables in Molecular Simulations with Flexible Constraints

**DOI:** 10.1371/journal.pone.0189454

**Published:** 2017-12-06

**Authors:** Pablo Echenique, Claudio N. Cavasotto, Monica De Marco, Pablo García-Risueño, J. L. Alonso

The fourth author’s name appears incorrectly. The correct name is: Pablo García-Risueño. The correct citation is: Echenique P, Cavasotto CN, De Marco M, García -Risueño P, Alonso J (2011) An Exact Expression to Calculate the Derivatives of Position-Dependent Observables in Molecular Simulations with Flexible Constraints. PLoS ONE 6(9): e24563. https://doi.org/10.1371/journal.pone.0024563
